# Efficacy and safety of sitagliptin for the treatment of diabetes mellitus complicated by chronic liver injury

**DOI:** 10.1186/s40064-015-1135-z

**Published:** 2015-07-15

**Authors:** Masahiro Asakawa, Hiroshi Mitsui, Momoko Akihisa, Tetsuo Sekine, Yoshihiro Niitsu, Arisa Kobayashi, Atsuko Miyake, Naoaki Hashimoto, Mitsunobu Kawamura, Yoshihiro Ogawa

**Affiliations:** Department of Endocrinology and Metabolism, Tokyo Teishin Hospital, 2-14-23, Fujimi, Chiyoda-ku, Tokyo, 102-0071 Japan; Department of Gastroenterology, Tokyo Teishin Hospital, 2-14-23, Fujimi, Chiyoda-ku, Tokyo, 102-0071 Japan; Department of Molecular Endocrinology and Metabolism, Tokyo Medical and Dental University Hospital, 1-5-45, Yushima, Bunkyo-ku, Tokyo, 113-8510 Japan

**Keywords:** Dipeptidyl peptidase-4 inhibitor, Sitagliptin, Diabetes mellitus, Chronic liver injury

## Abstract

**Aim:**

To investigate the efficacy and safety of a dipeptidyl peptidase-4 inhibitor, sitagliptin, for treating diabetes mellitus complicated by chronic liver injury.

**Methods:**

Sitagliptin was administered for 13.7 ± 10.1 months to 122 patients with DM complicated by chronic liver injury (including 19 patients with liver cirrhosis), and changes in hemoglobin A1c (HbA1c) and liver enzymes (transaminases, etc.) were evaluated.

**Results:**

HbA1c was reduced from 8.48 ± 1.43% to 7.87 ± 1.35% (P < 0.001). Among liver enzymes, alanine aminotransferase (ALT) levels improved from 75.1 ± 45.2 to 65.8 ± 35.8 IU/L (P = 0.012) and gamma-glut amyl-trans peptidase from 155.2 ± 161.1 to 133.2 ± 127.4 IU/L (P = 0.044). Among the causes of liver injury, non-alcoholic fatty liver disease and alcoholic liver disease both showed the reductions in HbA1c with no deterioration of liver enzymes. An analysis of 19 patients with liver cirrhosis also showed reductions in HbA1c with no deterioration of liver enzymes.

**Conclusion:**

It is suggested that sitagliptin can be administered effectively and safely to patients with diabetes mellitus complicated by chronic liver injury, including liver cirrhosis.

## Background

The number of patients with diabetes mellitus (DM) is increasing recently in Japan, was estimated at 9.5 million cases in 2012 in reports by Japanese Ministry of Health, Labor and Welfare. Among persons with probable DM, the rates of patients receiving medical treatment are 65.9% for men and 64.3% for women, percentages that remain far too low. Dipeptidyl-peptidase 4 (DPP-4) inhibitors have recently been developed as a new viable option for the treatment of DM other than for type 1 DM (T1DM), and the first DPP-4 inhibitor in Japan, sitagliptin, was released in 2009. Subsequently, a variety of DPP-4 inhibitors have been released, so that there were seven DPP-4 inhibitors available by 2013.

The incretin hormones glucagon-like peptide-1 (GLP-1) and glucose-dependent insulinotropic polypeptide (GIP) are released from the gastrointestinal tract in response to a meal, which stimulates glucose-dependent insulin production and secretion via specific receptors expressed on the islet β cells (Drucker 2002). DPP-4 inhibitors exhibit serum glucose-decreasing action by inhibiting the decomposition of incretin hormones and increasing of insulin secretion (Drucker and Nauck 2006; Ahren 2007; Holst et al. 2009; Omar and Ahren 2014). DPP-4 inhibitors have no effect on weight gain and pose little risk of hypoglycemia following a single administration, which are benefits that have resulted in recently increasing numbers of prescriptions. In addition, DPP-4 inhibitors inhibit glucagon secretion, increase islet cell proliferation, and decrease cell apoptosis in vitro (Farilla et al. 2002; Li et al. 2003). Since the existence of GLP-1 receptors has been confirmed in several organs other than pancreas, such as stomach, heart, kidney, liver, lung, central nervous system, muscle cells, fat cells, and vascular endothelial cells (Ahren 2004), extra-pancreatic effects of DPP-4 inhibitors are also expected.

Patients with type 2 diabetes mellitus (T2DM) patients often also present with chronic liver injury, and a previous study showed that approximately 80% of T2DM patients have a fatty liver (Browning et al. 2004). In particular, non-alcoholic fatty liver disease (NAFLD) is a frequent complication of T2DM (Arase et al. 2009), and is the most common form of chronic liver injury in many countries around the world (Angulo 2002). In addition, DM has been suggested to enhance the development of hepatocellular carcinoma in patients with chronic hepatitis C (Kawamura et al. 2008; Veldt et al. 2008). As stated above, DM is closely related to chronic liver injury. On the other hand, since most oral hypoglycemic agents are metabolized in the liver and may induce liver damage, the treatment of T2DM patients with chronic liver injury is often difficult (Nauck et al. 2007).

Since the DPP-4 inhibitor sitagliptin is minimally metabolized in the liver and over 80% is excreted in an unaltered state in the urine (Drucker and Nauck 2006), it is expected that the pharmacokinetic of sitagliptin will have few negative effects even in patients with chronic liver injury. Although previous studies have shown the efficacy and safety of sitagliptin in a few patients with NAFLD or chronic hepatitis C (Iwasaki et al. 2011; Arase et al. 2013; Arase et al. 2011), the investigations remain insufficient.

In this study, we investigated the efficacy and safety of sitagliptin in patients with non-T1DM complicated by chronic liver injury.

## Methods

### Patients

We investigated previous prescriptions written in Tokyo Teishin Hospital, and found 1,148 patients with DM other than T1DM who were administered sitagliptin (50 mg/day) between April 2010, and September 2013. Among them, the DM of 122 patients was complicated by liver injury at the start of sitagliptin administration. We regarded a patient to have liver injury based on the following clinical data: (1) elevation of serum aspartate aminotransferase (AST) and/or alanine aminotransferase (ALT) levels, or (2) the existence of primary liver disease associated with thrombocytopenia with platelet counts below 100,000/μL. The causes of liver injury were assessed comprehensively from past medical histories, drinking habits (daily alcohol consumption above or below 20 g), clinical data such as viral markers of hepatitis, liver fibrosis markers, antinuclear antibodies, and imaging views by abdominal ultrasonography and/or computed tomography (CT). NAFLD was diagnosed for patients with fatty liver alleged by CT and/or ultrasound, with daily alcohol consumption below 20 g, and with negative hepatitis B and C virus and autoantibodies. Alcoholic liver disease (ALD) was diagnosed for patients with elevated liver enzymes, with daily alcohol consumption above 20 g, and with negative hepatitis B and C virus and autoantibodies. Chronic hepatitis C (C–CH) was diagnosed for patients with positive hepatitis C virus markers; antibody for hepatitis C virus or positive hepatitis C virus’ RNA. Autoimmune hepatitis (AIH) was diagnosed for patient with positive anti-nuclear antibody, elevated immunoglobulin G (IgG), negative anti-mitochondrial antibody, and with chronic hepatitis characterized by interface hepatitis or infiltration of plasma cells confirmed by liver biopsy. Moreover, patients with liver cirrhosis were diagnosed based on findings of CT and/or ultrasound, which showed rough liver surface, rough echoic levels in liver, dull liver edge, splenomegaly, and ascites, and blood tests, which showed thrombocytopenia, and reduced biosynthetic capacity such as albumin, prothrombin, total cholesterol, and cholinesterase.

### Clinical and laboratory evaluation

We conducted retrospective investigation of the 122 patients based on the clinical data in their medical records at the start of sitagliptin administration (pre-treatment) and at the end of the study period (September 2013) (post-treatment). All included patients received sitagliptin for a minimum of 3 months. If sitagliptin administration was ceased for some reason prior to September 2013, the data at the time of cessation were used as the post-treatment values. Serum levels of AST, ALT, gamma-glut amyl-trans peptidase(γGT) and hemoglobin A1c (HbA1c, NGSP value) were compared pre- and post-treatment.

In addition, we investigated the clinical data classified by the type of chronic liver injury: the NAFLD group and ALD group. We also investigated the clinical data for the LC group independently.

### Statistical analysis

SPSS ver. 20 for Windows software was used to perform statistical analysis. Data are expressed as mean ± SD unless indicated otherwise. The paired *t* test was used to compare the pre-treatment and post-treatment clinical data. A *p* value <0.05 was considered statistically significant.

## Results

### Characteristics of enrolled patients

Table 1 shows the characteristics before follow-up of the 122 patients (93 men and 29 women: mean age, 58.5 ± 13.9) with DM complicated by chronic liver injury. Nineteen patients were diagnosed with LC, 10 with ALD, 6 with C–CH, 2 with NAFLD and one with AIH. Two patients with NAFLD were considered to develop non-alcoholic steatohepatitis (NASH), which shows the ballooning of hepatocytes, proliferation of inflammatory cells, and hepatic fibrosis in histology. LC patients were graded by their Child-Pugh score (Cholongitas et al. 2005), which suggests liver functional reserves, with 12 classified as Child-Pugh A, 5 as Child-Pugh B, and 2 as Child-Pugh C.

**Table 1:** Baseline characteristics

	Total	Non LC			LC
		NAFLD	ALD	C–CH	
n	122	62	17	3	19
Age (years)	58.5 ± 13.9	55.9 ± 15.4	60.1 ± 10.9	53.6 ± 14.8	66.4 ± 8.7
Sex (male/female)	93/29	48/14	16/1	0/3	13/6
Duration of diabetes (years)	9.2 ± 7.5	8.0 ± 5.6	9.0 ± 6.9	10.3 ± 9.5	9.0 ± 4.8
BMI (kg/m^2^)	26.9 ± 5.4	28.0 ± 5.3	26.8 ± 5.4	29.3 ± 15.8	23.9 ± 2.8
Platelet (×104/μL)	18.3 ± 6.5	20.3 ± 5.7	19.0 ± 4.6	20.5 ± 6.6	9.3 ± 4.0
HbA1c (%)	8.48 ± 1.43	8.57 ± 1.23	8.29 ± 1.77	9.76 ± 2.70	8.12 ± 1.29
AST (IU/L)	56.0 ± 25.8	52.7 ± 25.3	66.5 ± 28.3	71.0 ± 26.2	60.4 ± 30.9
ALT (IU/L)	75.1 ± 45.2	79.2 ± 45.4	88.0 ± 67.4	94.0 ± 10.8	49.5 ± 28.4
γGT (IU/L)	155.2 ± 161.1	112.0 ± 80.4	232.8 ± 144.7	92.3 ± 47.4	277.6 ± 310.4
Duration of sitagliptin dosing (months)	13.7 ± 10.1	15.2 ± 10.0	10.6 ± 9.1	7.3 ± 6.6	9.1 ± 9.6

The data represent the mean ± SD.

AIH in 1 patient and unknown cause in 20 patients in addition to the data above.

*HbA1c* hemoglobin A1c, *AST* aspartate aminotransferase, *ALT* alanin aminotransferase.

The causes of chronic liver injury in the remaining 103 patients (non-LC group) were NAFLD in 62 patients, ALD in 17 patients, C–CH in 3 patients and autoimmune hepatitis (AIH) in 1 patient. As one patient with AIH was treated by prednisolone, her DM was possibly exacerbated by steroid therapy. The causes of chronic liver injury in 20 patients were unknown due to insufficient clinical data.

Pre follow-up, 67 patients were receiving no oral hypoglycemic drugs; 25 patients were receiving one drug (biguanide (BG) in 9 patients, sulfonylurea (SU) in 8 patients, insulin in 7 patients, and thiazolidine (TZD) in one patient); 26 patients were receiving two drugs (SU and BG in 16 patients, insulin and BG in 3 patients, TZD and BG in 3 patients, insulin and SU in 2 patients, and SU and α-glucosidase inhibitor (α-GI) in 2 patients); 4 patients were receiving three drugs (insulin, BG and α-GI in 1 patient, insulin, SU and α-GI in 1 patient, SU, BG and TZD in 1 patient, and SU, BG and α-GI in 1 patient).

### Changes in clinical data

**Figure 1:**
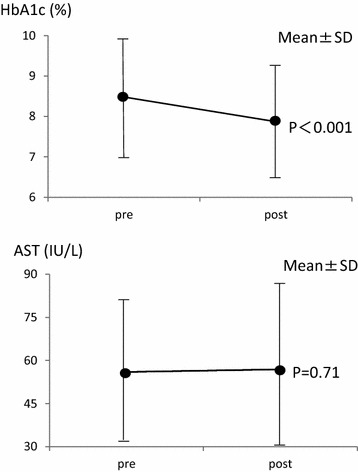
Changes in HbA1c and AST in all patients.

**Figure 2:**
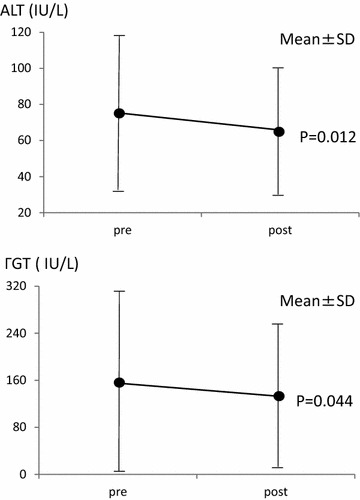
Changes in ALT and γGT in all patients.

The duration of sitagliptin administration was 13.7 ± 10.1 months on average. The average HbA1c levels decreased from 8.48 ± 1.43 to 7.87 ± 1.35% (P < 0.001). Among liver enzymes, average AST levels underwent no significant change (from 56.0 ± 25.8 IU/L to 56.9 ± 27.6 IU/L, P = 0.718), while average ALT levels decreased (from 75.1 ± 45.2 to 65.8 ± 35.8 IU/L, P = 0.012), and average γGT levels decreased (from 155.2 ± 161.1 to 133.2 ± 127.4 IU/L, P = 0.044) (Figures 1, 2).

As shown in Table 2, a reduction in HbA1c without any deterioration in liver enzymes was observed for patients whose liver injury was classified as either NAFLD or ALD (non-LC), although the reduction in HbA1c in the ALD group was not statistically significant. An analysis of patients with LC also showed a reduction in HbA1c without a deterioration in liver enzymes (Table 3).

**Table 2:** Changes in clinical data of diabetes mellitus complicated by chronic liver injury (NAFLD and ALD)

	NAFLD group (n = 62)				ALD group (n = 17)		
	Pre-treatment	Post-treatment	P value		Pre-treatment	Post-treatment	P value
HbA1c (%)	8.57 ± 1.23	7.99 ± 1.22	<0.001	HbA1c (%)	8.29 ± 1.77	7.79 ± 1.42	0.099
AST (IU/L)	52.7 ± 25.3	55.5 ± 29.4	0.491	AST (IU/L)	66.5 ± 28.3	63.1 ± 22.0	0.563
ALT (IU/L)	79.2 ± 45.4	74.7 ± 41.5	0.398	ALT (IU/L)	88.0 ± 67.4	63.0 ± 26.1	0.083
γGT (IU/L)	112.0 ± 80.4	115.4 ± 95.7	0.713	γGT (IU/L)	232.8 ± 144.7	163.8 ± 102.3	0.023

**Table 3:** Changes in clinical data of diabetes mellitus complicated by liver cirrhosis

	LC group (n = 19)		
	Pre-treatment	Post-treatment	P value
HbA1c (%)	8.12 ± 1.29	7.38 ± 1.24	0.006
AST (IU/L)	60.4 ± 30.9	64.5 ± 33.3	0.628
ALT (IU/L)	49.5 ± 28.4	44.5 ± 24.3	0.483
γGT (IU/L)	277.6 ± 310.4	212.7 ± 277.5	0.237

### Safety of sitagliptin

Three patients developed mild hypoglycemia (one patient in the NAFLD group receiving sulfonylurea, one patient in the ALD group receiving insulin, and 1 patient in the LC group receiving sulfonylurea), but no severe cases of hypoglycemia were seen in this study. Four patients died during the follow-up period; one was due to myeloproliferative disease (71 year-old male in the LC group), one was due to brain hemorrhage (77 year-old male in the LC group), one was due to pneumonia (88 year-old male in the NAFLD group), and one was due to rupture of a hepatocellular carcinoma (65 year-old male in the LC group). We cannot deny the relationship between rupture of a hepatocellular carcinoma and sitagliptin administration, because recently it has been demonstrated that CXCR4/CXCL12 axis exerts a variety of functions at different steps of hepatocellular carcinoma progression, and CXCL12 may be inactivated by DPP-4 cleavage (Ismael et al. 2014). No other adverse events developed, and no patients stopped sitagliptin treatment due to adverse effects.

## Discussion

We have described the efficacy and safety of sitagliptin as a treatment for patients with DM complicated by chronic liver injury. The present study shows that the administration of sitagliptin for an average of 13.7 months resulted in 0.61% decrease in the level of HbA1c, as well as decreased ALT and γGT levels in 122 patients including 19 patients with LC. The ALT and γGT levels did not decrease significantly when analyzed with respect to the causes of chronic liver injury, but the data suggest at least that no deterioration of AST, ALT and γGT levels was caused by sitagliptin. In particular, this study can be considered valuable in demonstrating that sitagliptin decreased HbA1c levels without causing any deterioration in liver enzymes even in patients with LC.

Recently, several studies have examined the relationships between DPP-4 or incretin hormones and liver. DPP-4 plays a role in fibroblast activation in the liver by activating hepatic stellate cells (Piazza et al. 1989; Levy et al. 1999), while sitagliptin, a DPP-4 inhibitor, attenuates hepatic fibrosis by suppressing activated hepatic stellate cells in rats (Kaji et al. 2014). The GLP-1 receptor is present on human hepatocytes and has been shown to play a direct role in decreasing hepatic steatosis in vitro by modulating components of the insulin signaling pathway (Gupta et al. 2010). GLP-1 suppresses hepatic lipogenesis by activating the cAMP-activated protein kinase (AMPK) pathway (Shlomo et al. 2011). Although the investigations in vivo are still insufficient, the data suggest that DPP-4 inhibitors and GLP-1 receptor agonists may improve hepatic steatosis and suppress the progression of hepatic fibrosis.

NAFLD is the leading cause of chronic liver injury in T2DM, as was seen in this study. NAFLD is considered to be a hepatic manifestation of the metabolic syndrome, and is particularly associated with insulin resistance, obesity, hypertension, and abnormalities in glucose and lipid metabolism (Chitturi et al. 2002). The histological changes seen in NAFLD range over a wide spectrum, extending from simple steatosis, which is generally non-progressive, to NASH, liver cirrhosis and liver failure, and, sometimes, even hepatocellular carcinoma (Ludwig et al. 1980). It is expected that DPP-4 inhibitors and GLP-1 receptor agonists will be especially effective in treating T2DM patients with NAFLD, because several studies have shown that the serum concentration of DPP-4 is high in NAFLD patients where it is related to insulin resistance (Firneisz et al. 2010), and that the expression of GLP-1 receptor is reduced in NASH patients (Ding et al. 2006). A previous study showed that the administration of sitagliptin (50 mg/day) for 16 weeks resulted in significant decreases in serum HbA1c, AST, ALT and γGT levels in 30 NAFLD patients with T2DM (Iwasaki et al. 2011), a finding that suggests sitagliptin improves the effects of fatty liver. Although significant decreases in the serum levels of AST, ALT and γGT were not found for 62 NAFLD patients in our study, further investigations are necessary to assess the efficacy of sitagliptin in NAFLD patients. Although our study included NAFLD patients co-treated with thiazolidine, which has the efficacy of improving liver histology and fibrosis in NASH patients (Boettcher et al. 2012), the conclusion has not been changed even if these patients were excluded from the analysis because the number of these patients was only 3.

We also investigated the efficacy and safety of sitagliptin also in ALD and LC patients. In particular, efficacy and safety of sitagliptin in LC patients have not been sufficiently investigated until now. LC can be defined as the end stage of chronic liver disease with progressive fibrosis, and can be fatal due to the development of hepatocellular carcinoma or liver failure (Bataller and Brenner 2005). It is often difficult to treat patients with T2DM complicated by liver diseases using oral hypoglycemic agents, because most existing oral hypoglycemic agents are metabolized in the liver, which may lead to an increase in hypoglycemic episodes or a deterioration of liver function. Since the DPP-4 inhibitor sitagliptin is only minimally metabolized in liver with over 80% excreted intact in urine (Drucker and Nauck 2006), it is expected that sitagliptin will reduce serum glucose levels without causing a deterioration in liver enzymes even in LC patients. This study investigated 19 patients with LC including those with severely impaired hepatic functional reserves (5 patients classified as Child-Pugh B and 2 patients as Child-Pugh C), with no apparent deterioration in liver enzymes. Although the number of patients is low, the result can be considered valuable. Since a previous study showed that sitagliptin attenuates hepatic fibrosis by suppressing activated hepatic stellate cells in vitro (Kaji et al. 2014), it is worthy investigating further whether sitagliptin suppresses the progression of hepatic fibrosis in LC patients.

Although we believe our findings to be valuable, the limitations of this study are as follows. First, this study is a retrospective, observational study, and the duration of sitagliptin administration was not the same in all cases. Second, the number of patients was insufficient (122 patients), and, moreover, the causes of chronic liver injury in 20 patients were unknown due to the lack of sufficient clinical data. Third, we only assessed liver enzymes such as serum AST, ALT and γGT levels, and the assessments for true liver function, such as serum albumin, prothrombin, cholinesterase and bilirubin, are insufficient. Fourth, the degree of hepatic fibrosis was not assessed using objective markers such as serum hyaluronic acid or type IV collagen 7S domain, or other modalities such as Fibroscan. Further investigations are necessary to assess efficacy and safety of sitagliptin for the treatment of patients with DM complicated by chronic liver injury.

## Conclusion

It is suggested that sitagliptin can be administered effectively and safely to patients with diabetes mellitus complicated by chronic liver injury, including those with liver cirrhosis.

## Abbreviations

DM: diabetes mellitus
T2DM: type 2 diabetes mellitus
DPP-4: dipeptidyl peptidase-4
GLP-1: glucagon-like peptide-1
GIP: glucose-dependent insulinotropic polypeptide
NAFLD: non-alcoholic fatty liver disease
ALD: alcoholic liver disease
C–CH: chronic hepatitis C
NASH: non-alcoholic steatohepatitis
LC: liver cirrhosis
AIH: autoimmune hepatitis
SU: sulfonylurea
BG: biguanide
TZD: thiazolidine
α-GI: α-glucosidase inhibitor
HbA1c: hemoglobin A1c
AST: aspartate aminotransferase
ALT: alanine aminotranseferase
γGT: gamma-glut amyl-trans peptidase
